# Composition and pathogenic potential of a microbial bioremediation product used for crude oil degradation

**DOI:** 10.1371/journal.pone.0171911

**Published:** 2017-02-08

**Authors:** Azam F. Tayabali, Gordon Coleman, Jennifer Crosthwait, Kathy C. Nguyen, Yan Zhang, Philip Shwed

**Affiliations:** Biotechnology Laboratory, Environmental Health Science and Research Bureau, Healthy Environments and Consumer Safety Branch, Environmental Health Centre, Health Canada, Ottawa, Canada; Universita degli Studi di Milano-Bicocca, ITALY

## Abstract

A microbial bioremediation product (MBP) used for large-scale oil degradation was investigated for microbial constituents and possible pathogenicity. Aerobic growth on various media yielded >10^8^ colonies mL^-1^. Full-length 16S rDNA sequencing and fatty acid profiling from morphologically distinct colonies revealed ≥13 distinct genera. Full-length 16S rDNA library sequencing, by either Sanger or long-read PacBio technology, suggested that up to 21% of the MBP was composed of *Arcobacter*. Other high abundance microbial constituents (>6%) included the genera *Proteus*, *Enterococcus*, *Dysgonomonas* and several genera in the order *Bacteroidales*. The MBP was most susceptible to ciprofloxacin, doxycycline, gentamicin, and meropenam. MBP exposure of human HT29 and A549 cells caused significant cytotoxicity, and bacterial growth and adherence. An acellular MBP filtrate was also cytotoxic to HT29, but not A549. Both MBP and filtrate exposures elevated the neutrophil chemoattractant IL-8. In endotracheal murine exposures, bacterial pulmonary clearance was complete after one-week. Elevation of pro-inflammatory cytokines IL-1β, IL-6, and TNF-α, and chemokines KC and MCP-1 occurred between 2h and 48h post-exposure, followed by restoration to baseline levels at 96h. Cytokine/chemokine signalling was accompanied by elevated blood neutrophils and monocytes at 4h and 48h, respectively. Peripheral acute phase response markers were maximal at 24h. All indicators examined returned to baseline values by 168h. In contrast to HT29, but similar to A549 observations, MBP filtrate did not induce significant murine effects with the indicators examined. The results demonstrated the potentially complex nature of MBPs and transient immunological effects during exposure. Products containing microbes should be scrutinized for pathogenic components and subjected to characterisation and quality validation prior to commercial release.

## Introduction

Microorganisms are used in biotechnology processes for a variety of industrial, consumer and environmental applications. One such application is the *in situ* degradation of environmental contaminants that accumulate over time from pollution or as a result of accidental spills [1–7]. One approach for these applications is *in situ* degradation with microbial bioremediation products (MBPs), which involves the dissemination of microorganisms into contaminated soil or water ecosystems. The degradation of the contaminant can occur directly by one or more microorganisms or through the synergism of a mixture of microbes, whether deliberately formulated or naturally occurring (i.e., a consortium). Dominant species then emerge as a result of nutrient selection that are capable of metabolizing the contaminant into a less harmful state.

As with any substance, the health risk associated with a microbial-based industrial or consumer product will be dependent on the type and number of microbial species present and the probability of exposure. For the latter, occupational exposure through ingestion or inhalation during the manufacture and application of the product may be most significant. Currently, there is some information on the toxicity associated with microorganisms used in biotechnology applications [8–11], but none for MBPs. Besides a lack of empirical data on the toxicity of MBPs, several other factors confound assessing their potential hazards. The final formulation may consist of non-biological components that enhance their efficacy and possibly their toxicity. In products containing combinations of living microorganisms, the contents may continually change over time as some species out-compete others under different conditions encountered during manufacturing, storage, transportation and in situ bioremediation. Furthermore, MBPs may vary from lot to lot, and may contain unintended living and non-living constituents or contaminants that could contribute to toxicity that may also vary between lots. As such, the hazard evaluation of the microbial product will depend on the stability and lot-to-lot variation of the preparations.

Given these challenges and uncertainties, there is interest in developing a systematic methodology to analyse complex MBPs. We selected as a model MBP an industrial product intended for *in situ* or *ex situ* degradation of soil and water contaminated with petroleum hydrocarbon. Furthermore, this product was chosen since some genus-level identification had already been conducted [12]. The contents of this product was further analysed using both growth-dependent and DNA sequencing methods. Furthermore, the potential hazard of the entire MBP was assessed using standardized mammalian cytotoxicity and murine inhalation exposure assays. Overall, the results demonstrated that the constituents of the MBP were complex, and provided evidence for transient but significant inflammation that may result during exposure.

## Materials and methods

### Viability of the MBP

A model MBP was obtained from an anonymous commercial supplier in 55-gallon containers. The product is used for the biodegradation of petroleum hydrocarbon contaminated soil and water, and is described by the manufacturer as containing selected, naturally occurring, non-pathogenic microorganisms that were genetically unaltered. Two lots were purchased approximately one year apart. A sample of the MBP can be provided to interested researchers on request. Please contact the corresponding author. The MBPs were stored according to the manufacturer’s recommendations at room temperature. The first lot was stored for 4 weeks prior to characterisation over a period of 20 weeks. The second lot was stored for one week and characterised for 12 weeks. The microbial content of the commercial product was quantified twice a week by spread-plating serial dilutions onto different selective agar media incubated at room temperature and 37°C. Plates were examined for colony formation daily for one week. Selective agars to assess growth included cetrimide, citrate, Czapek, Dulbecco’s modified Eagle’s Medium (DMEM), Luria-Bertani, lysine-iron, Mycosel, potato dextrose, Sabouraud’s, sheep blood, sheep blood base, triple sugar—iron, trypticase soy, urea, and yeast malt.

### Identification of cultivable microbes

For fatty acid phenotyping, the procedures outlined by MIDI-Sherlock^®^ fatty acid methyl ester (FAME) microbial identification system (MIDI Inc., DE) were followed. Samples prepared for comparisons to the aerobic environmental bacterial library (RTBSA6) were cultured on trypticase soy broth (TSB) agar for 24 h at 28°C. Samples prepared for the aerobic clinical library (RTCLIN6) were cultured on Tryptic Soy Agar with 5% sheep blood for 24 h at 37°C. Whole cell fatty acids were saponified, methylated, and extracted as described in the MIDI identification system operating manual. Extracted FAMEs were separated with an Agilent Technologies series 6890N gas chromatography system equipped with a 25 m x 0.2 mm crosslinked 5% phenyl methyl silicone fused silica capillary column. Analysis was based on calibration retention times determined prior to sample analysis. The Sherlock^®^ sequencer software version 4.5 and Sherlock^®^ microbial identification software version 6.1 were used to identify each chromatography feature, and compare the FAME profiles to those of known microorganisms in the environmental and clinical libraries.

For genetic identification of colonies, the MBP was serially diluted and spread-plated on various selective media. DNA from each distinct colony was extracted using the Masterpure^™^ DNA isolation kit (Epicentre^®^, WI). Full-length 16S ribosomal DNA (rDNA) was amplified using universal primers 24F (5’TGGAGAGTTTGATCCTGGCTCAG3’) and 1492R(5’ACCTTGTTACGACTT3’) using LA Taq polymerase (Clonetech Laboratories, ON) and the following conditions: 30 cycles; 94°C for 30 sec, 49°C for 30 sec, 72°C for 2 min. Amplicons were sequenced on an Applied Biosystems 3130xl genetic analyzer using BigDye terminator v3.1 sequencing chemistry (Life Technologies, ON). Sequences were compared to public (Ribosomal Database Project build 11; http://rdp.cme.msu.edu) and proprietary (Microseq^™^ Microbial ID full length 16S rDNA library v2.0, Applied Biosystems) databases for classification purposes.

### Metagenomic analysis

Metagenomic DNA (mDNA) was isolated directly from cryopreserved (-80°C, 10% glycerol) aliquots of the MBP batches using the Powerlyzer Powersoil gDNA Isolation kit (MoBio Labs, Carlsbad, CA). Approximately 200 ng template mDNA was amplified with bacterial 16S rDNA primers 24F (5’ TGGAGAGTTTGATCCTGGCTCAG-3’) and 1492R (5’-GGTTACCTTGTTACGACTT-3’) primers and PCR amplicons were cloned into pCR2.1 vector (Life Technolgies, ON), transformed into DH5α competent *E*. *coli* and scaled up for library generation. Transformants were then plated on selective agar and randomly picked for sequencing. Plasmid DNA was isolated by alkaline lysis miniprep (Qiagen) and clones were sequenced using universal 16S primers and classified as above using an Applied Biosystems 3130 Genetic Analyzer and BigDye^®^ v 3.1 Terminator Cycle Sequencing Kit (Life Technologies, ON). Metagenomic DNA was also amplified with primers for the fungal large subunit variable D2 region PCR kit (Life Technologies, ON) and sequenced directly using the Microseq D2 Sequencing kit (Life Technologies) or used as above for library construction.

Full length 16S rDNA was also amplified from MBP mDNA (100 ng) using 16S rRNA gene primers 24F and 1492R using gradient PCR conditions as described by [13]. The resulting pooled amplicons were ligated with SMRTbell adapters and sequenced on a PacBio RSII sequencing system (Pacific Biosciences, CA). Raw sequencing data was converted to high quality circular consensus sequences (CCS) using the PacBio SMRTAnalysis software suite (version 2.2.0) which gave a total of 135,856 raw reads. Data processing was done as described by Tremblay et al., 2015 and relative abundance was determined using the QIIME software suite (version 1.8.0) [14].

### Antibiotic Minimal Inhibitory Concentration (MIC) assay

Antibiotic susceptibility and determination of minimal inhibitory concentration (MIC) was done using two methods. Agar plates separated by quadrants containing either no antibiotic, gentamicin (500 μg mL^-1^), streptomycin (2000 μg mL^-1^), or vancomycin (6 μg mL^-1^) (Synergy Quad, Remel Products, Thermo Fisher Scientific, KS) were inoculated with 300 μL of MBP and examined after 48 h at room temperature. The broth dilution method was done according to Tayabali and colleagues [11,15]. Antibiotics (Sigma-Aldrich Canada, ON) were amoxicillin, aztreonam, ceftazidime, ciprofloxacin, doxycycline, erythromycin, gentamicin, and meropenem, trimethoprim and vancomycin. A matched set of wells contained Amphotericin B to determine the contribution of fungi. Antibiotics diluted in TSB were pipetted into 96-well plates, each well with a final concentration of 0, 0.38 0.75, 1.5, 3, 6, 12, or 24 μg ml^-1^. After 24 h at 37°C, one mg mL^-1^ of 3-(4,5-dimethylthiazol-2-yl)-2,5-diphenyl tetrazolium bromide (MTT; Sigma-Aldrich) was added to each well, and plates were incubated for an additional two hours. The MIC value was defined as the minimum value for which there was no biological conversion (i.e., bioreduction) of MTT into formazan.

### Mammalian cell exposure effects

Human HT29 colonic epithelial cells (HTB-38) and A549 lung epithelial cells (CCL-185) were obtained from the American Type Culture Collection (Rockville, MD). HT29 cells were maintained in DMEM with 25 mM glucose with and 10% FBS with 50 μg mL^-1^ gentamicin whereas the A549 cells were maintained in F12K medium and 10% FBS with 100 U/mL penicillin G and 100 μg/mL streptomycin sulfate. Cells were exposed to bacteria as described previously [9–11]. HT29 and A549 cells (~80% confluent) were exposed to dilutions of MBP for various times in 96-well plates. Following exposure, the supernatant was removed and retained for multiplex measurement of IL-6, IL-8, G-CSF, GM-SCF, IFN-γ, TNF-α using an antibody-based bead array system (Bioplex^™^, BioRad, Mississauga, ON). Lipopolysaccharide (LPS) from *Pseudomonas* or *Escherichia* (Sigma-Aldrich) was used as a positive control for HT29 cytokine measurements. Cytotoxicity was initially measured with the MTT assay (Sigma-Aldrich). For this, fresh media with one mg mL^-1^ MTT was added to the wells and the plates were incubated for two hours at 37°C. Wells were rinsed twice with PBS to remove non-adherent cells and bacteria. Dimethyl sulfoxide (Sigma-Aldrich) was added to each well and the resultant solubilized-formazan was measured at OD_505_. The capacity of cells to reduce MTT to formazan was expressed as a percentage Bioreduction Activity compared to that of PBS-treated HT29 or A549. Alternatively, cell viability was measured with a Trypan blue dye exclusion method by using the Countess^™^ automated cell analyzer (Invitrogen, Burlington, Canada).

### Animal exposures

Animal exposures were done according to a previous publication [11]. Female Balb/c mice (Charles River Laboratories Inc., Saint-Constant, Quebec) between 18 and 23g were pair housed and were acclimated for one week and had access to food and sterile water *ad libitum*. Prior to endotracheal instillation, animals were lightly anesthetized with isoflurane. A dose of 1.25x10^7^ cfu 25 μL^-1^ saline was nebulized into the lungs with a Microsprayer^™^ (Penn Century, Philadelphia, PA). Treated mice were monitored to ensure recovery within two minutes. Six animals were treated for each treatment and time-point examined post-exposure, including a vehicle control group treated with saline. All aspects of the animal studies were approved by the Health Canada Animal Care Committee and overseen by a veterinarian.

### Blood analyses

As summarized in a previous publication [11], mice were lightly anaesthetized with isoflurane and exsanguinated by cardiac puncture. Blood was immediately placed into tubes containing ethylenediaminetetraacetic acid (EDTA) and gently mixed at room temperature. For differential leukocyte counts, blood was diluted 1:1 with Beckmann-Coulter diluent and loaded into a Beckmann-Coulter Ac.T 5-Diff^™^ Haematology Analyser. The remainder of the blood was centrifuged at 1000 xG for 10 min to prepare plasma, which was stored at -80°C for ELISA measurement of fibrinogen and SAA as described in protocols included by the kit manufacturer (Biosource International Inc., Camarillo, CA).

### Lung tissue processing

To measure bacterial pulmonary clearance, animals were euthanized at 2, 4, 24, 48, 96 and 168 h by cervical dislocation. Lungs were excised and homogenized in one mL of saline with a PowerGen 125 (Fisher Scientific). Lung homogenates were plated on Luria-Bertani -agar plates and bacterial colonies were enumerated after 18h at 37°C.

Pro-inflammatory cytokines, IL-1β, IL-6, IL-12(p70) and TNF-α, and chemokines KC and MCP-1, were measured using a Bioplex^™^ instrument. Lung portions were homogenised with a PowerLyzer 24 (MoBio Laboratories Inc., Carlsbad, CA). Multiplex cytokines/chemokines detection was done using bead kits (BioRad, Mississauga, ON) and a Bioplex 100 or 200 (BioRad), and data was analysed using Bioplex^™^ system software and Microsoft Excel^™^.

### Statistical analysis

Data analysis and graphing was done using Microsoft Excel^™^. Statistics was done with Sigmaplot version 11. Statistical significance between treatments was evaluated by analysis of variance (ANOVA) and either Tukey’s or Dunnett’s Multiple Comparison Test.

## Results

### MBP content determined by colony analysis

Two lots of industrial product were purchased from the supplier approximately one year apart. The first lot was stored for four weeks prior to initial sampling for numbers of cfu’s. A 90% drop in viability of the MBP (as measured by colony counts) was observed after the first six weeks when monitored over a five-month period. Toxicological analysis of this first lot was not pursued because of the rapid reduction in viability. The second lot was carefully monitored weekly following receipt, and all experiments, including metagenomic analyses, antibiotic assays, cytotoxicity, and murine exposures, were conducted within the first four weeks of receipt.

Several different colony morphologies were observed using various selective agars. Table 1 summarises some distinguishing colony characteristics and the numbers of colonies obtained. The greatest number of cfu’s were observed on 5% sheep blood, triple sugar, and starch agars, which corresponded to ~10^8^ cfu mL^-1^ in the original formulation. Identification of selected colonies was done by sequence comparison of the full length 16S rDNA sequence with those available through Microseq^™^ and RDP databases, and cellular fatty acid analysis using Sherlock^®^ MIDI-FAME^™^ libraries. In several instances, there was an agreement between colonies identified using the three methods.

**Table 1:** Identification of selective agar isolates (10 days at 21°C).

Selective Agar (cfu mL^-1^)	Distinguishing Colony Characteristics	16S rDNA RDP Identification (Similarity Score)^A^	16S rDNA Microseq Identification (% Identity)	FAME Identification (Similarity Index)^B^
Citrate (2.8 x 10^5^)	light grey dark blue	Enterobacter species (1.000) Citrobacter koseri (0.997)	Enterobacter asburiae (99.7) Citrobacter koseri (99.5)	NA^C^ Enterobacter cloacae (0.892)
MacConkey (2 x 10^7^)	mucoid, grey mucoid, yellow/red (Oxoid) yellow (Oxoid) yellow/red (Oxoid); positive (Difco) fine film appearance	Citrobacter amalonaticus (0.998) Klebsiella pneumonia (1.000) Ochrobactrum anthropi (1.000) Escherichia coli (0.999) Swine fecal bacterium (0.994)	Citrobacter amalonaticus (99.8) Klebsiella pneumonia (99.9) Ochrobactrum anthropi (99.5) Escherichia coli (99.5) Proteus penneri (99.2)	Escherichia coli (0.882) Proteus vulgaris (0.669) Ochrobactrum anthropi (0.994) Escherichia coli (0.562) NA
Mannitol (1.9 x 10^4^)	white yellow/tan	Jeotgalicoccus species (0.995) Arthrobacter creatinolyticus (0.995)	Macrococcus caseolyticus (91.6) Arthrobacter protophormiae (98.3)	Bacillus alcalophilus (0.456) Arthrobacter globiformis (0.494)
Sabouraud (6 x 10^7^)	abundant off-white mucoid	Citrobacter or Enterobacter (0.965) Enterococcus sanguinicola (0.996)	Enterococcus durans (99.2) Leclercia adecarboxylata (99.0)	Enterococcus mundii (0.419) NA
5% Sheep Blood (1.8 x 10^8^)	Hemolytic, grey hemolytic, white/cream weakly hemolytic, small weakly haemolytic, greyish non-hemolytic, small, dark hemolytic	Bacillus cereus/thuringiensis (1.000) Aquamicrobium lusatiense (0.997) Enterococcus avium (0.930) Escherichia coli (1.000) Enterococcus species (0.998) Enterococcus durans (1.000)	Bacillus thuringiensis (99.8) Labrys monachus (96.0) Erysipelothrix rhusiopathiae (97.5) Escherichia fergusonii (99.8) Enterococcus saccharolyticus (97.2) Enterococcus durans (99.2)	Bacillus cereus (0.550) Ochrobactrum anthropic (0.567) Enterococcus avium Escherichia coli (0.554) NA Enterococcus mundii (0.419)
100% Sheep Blood (4.8 x 10^7^)	non-hemolytic, shiny grey hemolytic, grey small colonies weakly haemolytic, greyish non-hemolytic, small, red non-hemolytic, shiny, grey	Bacillus species (0.999) Bacillus cereus (1.000) Enterococcus species (0.995) Bacillus cereus (1.000) Paracoccus solventivoran (0.998) Ochrobactrum species (0.999)	Bacillus fusiformis (96.2) Bacillus thuringiensis (99.8) Pseudomonas oleovorans (95.5) Bacillus cereus (99.9) Filomicrobium fusiforme (96.6) Ochrobactrum anthropic (99.5)	Bacillus GC group 22 (0.383) Bacillus cereus (0.550) NA NA NA Ochrobactrum anthropic (0.994)
Starch (1.6 x 10^8^)	white small colonies cream colored abundant radial pattern, ovoid shaped, small white radial pattern, ovoid shaped yellow	Bacillus or Lysinibacillus (0.980) Enterococcus species (0.998) Paracoccus solventivorans (0.998) Enterococcus species (0.999) Vavococcus fluvialis (1.000) Bacillus species (0.999) Proteus vulgaris (0.998) Arthrobacter creatinolyticus (0.978)	Bacillus fusiformis (96.2) Enterococcus durans (99.2) Filomicrobium fusiforme (95.9) Enterococcus saccharolyticus Vagococcus fluvialis (99.9) Bacillus fusiformis (99.5) Proteus penneri (99.2) Arthrobacter protophormiae (96.8)	Bacillus GC group 22 (0.383) Enterococcus mundii (0.419) NA NA NA Bacillus sphaericius (0.673) NA Arthrobacter globiformis (0.604)
Triple Sugar Iron (1.8 x 10^8^)	small abundant wave-like growth	Enterococcus avium (0.999) Enterococcus species (1.000) Proteus species (1.000)	Erysipelothrix rhusiopathiae (97.5) Enterococcus durans (99.2) Proteus penneri (99.2)	Enterococcus avium Enterococcus mundii (0.419) NA
Trypticase Soy Broth (8.8 x 10^7^)	grey off-white abundant, small grey abundant, white, convex abundant	Bacillus cereus (1.000) Lysinibacillus fusiformis (0.999) Enterococcus species (1.000) Enterococcus species (1.000) Enterococcus sanguinicola (0.999)	Bacillus thuringiensis (99.8) Bacillus fusiformis (99.95) Enterococcus raffinosus (98.6) Enterococcus durans (99.2) Enterococcus durans (99.1)	Bacillus cereus (0.550) Bacillus sphaericus (0.906) Enterococcus faecium (0.283) Enterococcus mundii (0.419) Suttonella (0.346)

^A^Similarity Score is the pairwise sequence identity calculated using a pairwise sequence alignment

^B^Similarity Index is the closeness of the bacterial fatty acid profile relative to the MIDI library entries

^C^NA—Data not available.

### Metagenomic analysis

**Fig 1:**
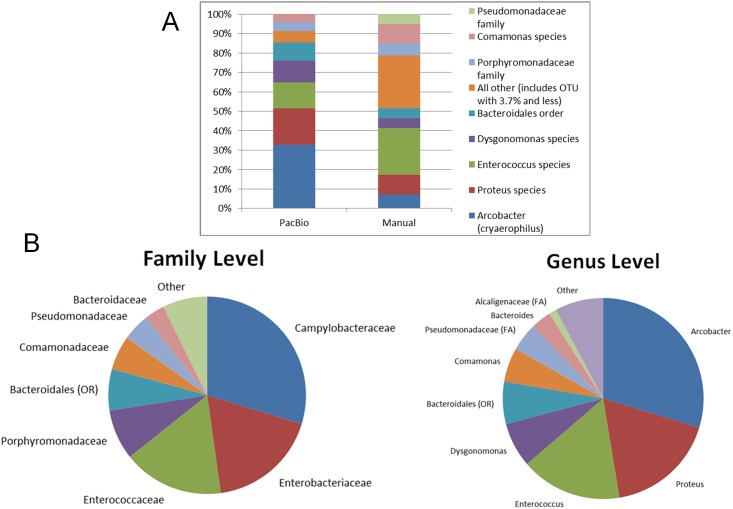
Taxonomic diversity and phylogenetic classification of MBPs. (A) Taxonomic diversity comparison of metagenomic data derived by Sanger sequencing of cloned 16S rDNA fragments (Manual; n = 103) compared with data derived from PacBio circular consensus sequencing (PacBio; n = 95,418) using the same taxonomic assignments. (B) Summary of phylogenetic classification at the family and genus level derived by PacBio circular consensus sequencing.

In order to reveal the composition and abundance of microbes in the MBP, clones from a library containing amplified full length 16S rDNA fragments derived from MBP metagenomic DNA were sequenced. Initial examination revealed that it was highly complex (Fig 1A, Manual lane). To clarify microbial diversity further, a large scale analysis involving long-read sequencing of a 16S rDNA amplicon was performed. A total of 135,856 Circular Consensus (CCS) reads were obtained using PacBio SMRTAnalysis (version 2.2.0) software. After removal of low quality reads and scanning for chimeras, a total of 110,985 CCS were kept and clustered at 97% ID (DNAclust version 3),which gave a total of 94,418 reads representing 44,919 clusters. Representative sequence clusters were classified with the RDP classifier (version 2.5) [16] with an in—house training set containing the Greengenes database [17]. Fig 1A shows relative taxon abundance with different proportions of the major constituents (version 1.8.0) [14]. By the PacBio methodology, the MBP appears to contain particular microbial constituents at relatively high abundance: *Arcobacter cryaerophilus* (21,3%), *Proteus* sp. (12.2%), *Enterococcus* sp (8.7%)., *Dysgomonas* sp. (7.2%) and *Bacteriodales* (6.1%).

PacBio sequence data (Fig 1B) showed that the most represented families in the MBP appeared to be the *Campylobacteraceae* (29.7%), *Enterobacteraceae* (18.1%), *Enterococccaee* (16.5%), *Porphyromonadaceae* (8.2%). The most abundant genera appeared to be *Arcobacter* (29.7%), *Proteus* (17.7%), *Enterococcus* (16.3%) and *Dysognomonas* (7.2%). Species level identifications showed the presence of *Bacteroides coprosuis* (3.5%), *Enterococcus asini* (2.2%), *Clostridium intestinale* (0.2%), and *Arcobacter cryaerophilus* (21.3%).

Additionally, an approximate assessment of fungal content was made by amplification and sequencing of the fungal D2 region directly from metagenomic DNA template. At least two fungal species appear present in the MBP, as identified by Microseq ID fungal database matches: *Aspergillus* (*niger*/*awamorii*/*foetidus*) (99.7% identity) and *Pseudoallescheri ellipsoidea* (99.2% identity).

### Antibiotic susceptibility analysis

**Fig 2:**
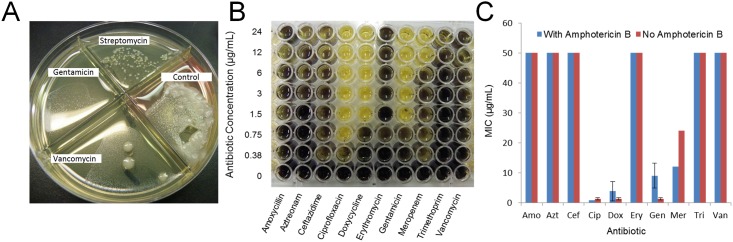
Antibiotic susceptibility. Two different antimicrobial assay formats were tested. Figure A shows resulting microbial growth on quadrant plates (Remel Synergy Quad^®^) containing BHI-agar with no antibiotic, 500 μg mL^-1^ gentamicin, 2000 μg/mL streptomycin, and 6 μg mL^-1^ vancomycin. Figs B and C demonstrate a colorimetric dilution method using MTT to highlight viable microorganisms. Each data point represents the mean of four separate experiments ± standard deviation.

In order to determine the antibiotic susceptibility of the MBP, two antimicrobial assays were tested. The MBP was inoculated into quadrant plates containing BHI media-agar with either no antibiotics, 500 μg mL^-1^ gentamicin, 2000 μg mL^-1^ streptomycin, or 6 μg mL^-1^ vancomycin (Fig 2A). All quadrants showed some growth, but notably less than the control containing no antibiotic. The broth dilution method (Fig 2B and 2C) demonstrated that the entire MBP was susceptible to ciprofloxacin, doxycycline, gentamicin, and meropenam. Amphotericin B had negligible influence on the susceptibility of the MBP to antibiotics.

### Cellular metabolic activity and pro-inflammatory cytokine effects

**Fig 3:**
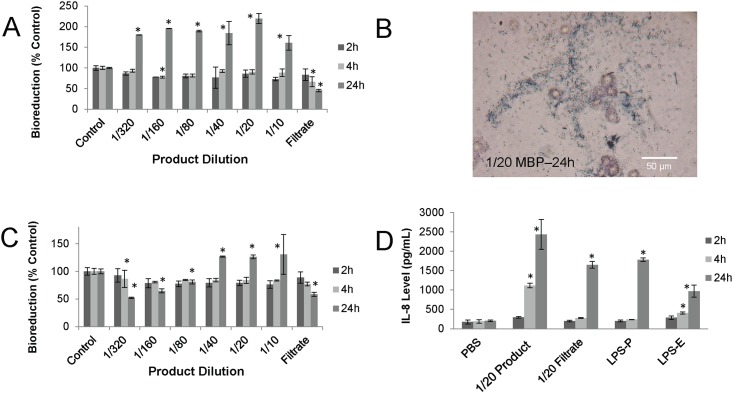
HT29 cytotoxicity and cytokine response. HT29 cells were exposed to 10^6^ cfu in 100 μL of MBP or its filtrate (1/20 dilution) either in the absence (A) or presence (B) of the broad-spectrum antibiotic, gentamicin. At various times, HT29 cells were assessed for metabolic activity using the MTT bioreduction assay. Supernatants from these exposures were tested for levels of pro-inflammatory cytokines and chemokines. (C) Photomicrograph demonstrating the adherence of bacteria to HT29 cells and the culture well surface. (D) IL-8 was found to be significantly elevated in response to some exposures in comparison to LPS positive controls isolated from *Pseudomonas* (LPS-P) and *Escherichia* (LPS-E). Each data point represents a representative experiment done in duplicate ± standard deviation. Asterisks indicate statistically different values compared to saline-treated controls (p<0.05).

HT29 colonic and A549 lung epithelial cells were used to assess the toxicity of the MBP. HT29 cells were exposed to dilutions of the MBP and assessed for cellular metabolism (i.e., bioreduction activity and trypan blue viability). Fig 3A shows that in the absence of antibiotic, minimal toxicity was observed with any of the treatments at two and four hours of exposure. In contrast, 24h exposure resulted in increases of 160 to 220% of control bioreduction at all MBP dilutions tested. This corresponded to exposures that contained significant microbial growth as observed by microscopy of the exposure wells (Fig 3B).

When using gentamicin to control bacterial contribution, only the 24h exposure with the most diluted MBP (1/320 and 1/160) induced a significant drop in HT29 bioreduction (Fig 3C). Surprisingly, an elevation above control bioreduction was also observed with exposures to concentrations at or above 1/40. Microscopy observations confirmed that there was still some microbial growth observed at these high microbial concentrations.

The MBP was filtered through a 0.22 μm pore size to determine the contribution of other components within the formulation such as lysed bacterial components and metabolites, as wells as formulation additives. Exposure to a 1/20 dilution of this filtrate demonstrated that although no microbial growth was apparent, cytotoxicity was observed at 4h (67% of control) and 24h (45% of control).

During the exposures, the HT29 supernatants were assessed for production of several cytokines and chemokines. Fig 3D shows that at 1/20 dilution, a significant increase in IL-8 was observed in the presence of gentamicin. No changes in the levels of other pro-inflammatory cytokines, IL-6, G-CSF, GM-CSF, IFN-γ, and TNF-α were observed and no changes were detected when antibiotic was omitted from the exposures. Positive controls, LPS from *Pseudomonas* or *Escherichia*, also caused a statistically significant elevation in IL-8 (Fig 3D).

**Fig 4:**
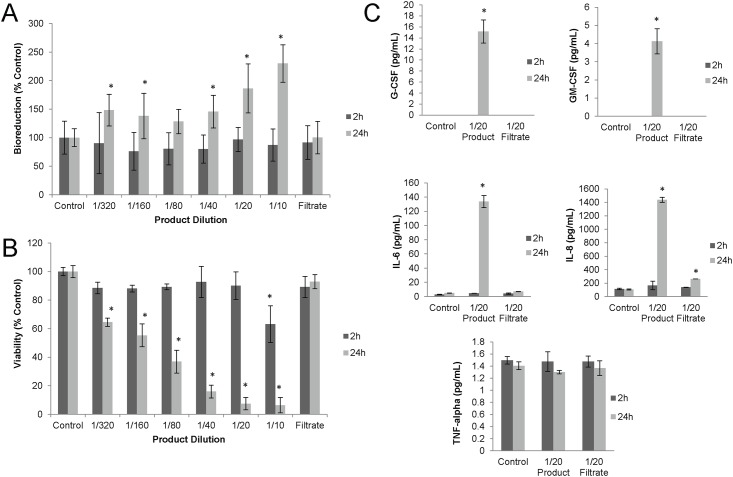
A549 cytotoxicity and cytokine response. A549 cells were exposed to dilutions in of MBP or its filtrate (1/20 dilution) either in the absence (A and B) or presence (C) of the broad-spectrum antibiotic, gentamicin. At various times, A549 cells were assessed for metabolic activity using the MTT bioreduction (A) or the Trypan Blue dye exclusion (B) assays. Supernatants from these exposures were tested for levels of pro-inflammatory cytokines and chemokines. (C) Cytokines and chemokines were found to be significantly elevated in response to some exposures. Each data point represents a representative experiment done in duplicate ± standard deviation. Asterisks indicate statistically different values compared to saline-treated controls (p<0.05).

A549 lung epithelial cells showed a similar elevation in MTT bioreduction activity at two and 24 h exposure to MBP (Fig 4A). In an effort to eliminate this microbial interference observed with the MTT assay, the trypan blue dye exclusion assay was used with A549 cells. Results presented in Fig 4B demonstrated that a dose-responsive reduction in viability (cytolysis) was observed with all MBP dilutions at 24h, but only with the 1/10 dilution at 2h exposure. Furthermore, unlike HT29 cells, A549 were not sensitive to filtrates, as viability following filtrate exposure was comparable to that of control cells.

A549 cells also demonstrated significant elevations of pro-inflammatory cytokines G-CSF, GM-CSF, IL-6 and chemokine IL-8, but IFN-γ was below the detection limit (not shown) and TNF-α levels were unchanged during exposure (Fig 4C). Again, in contrast to the HT29 result, no elevation of cytokines or chemokines resulted from A549 exposed to filtrate.

### Balb/c endotracheal exposures

**Fig 5:**
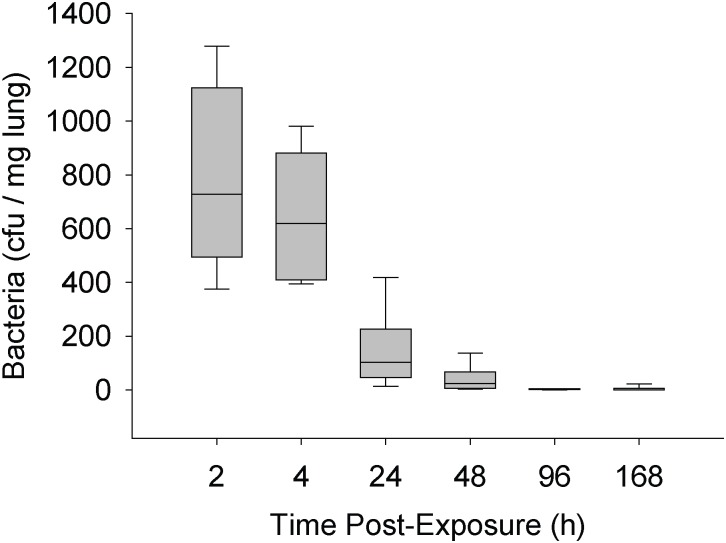
Pulmonary clearance of the MBP from Balb/c mice. Mice were exposed to 1.25x10^7^ cfu in 25 μL of MBP. At various times post-exposure, a subset of animals was euthanized. Portions of lungs were excised, homogenized, and spread plated onto LB-agar plates. The numbers of resulting microbial colonies after 24h incubation is shown. The box-whisker plots show the following: The horizontal line within each box represents the median from six animal treatments. Lower and upper boundaries of the boxes represent the 25th and 75th percentiles, respectively. The whiskers represent the 10th and 90th percentiles.

Female Balb/c mice were endotracheally exposed to the MBP at a concentration of 1.25 x 10^7^ cfu in 25μL. Exposed mice appeared symptomatically normal compared to saline-exposed controls; only showing some ruffled fur at 24–48h post-exposure. Mice were necropsied at various times post-exposure and various tissues were harvested for analysis. Pulmonary clearance of the bacteria was assessed by spread-plating lung homogenates and colony enumeration after 24h. Fig 5 demonstrates that the lungs were rapidly cleared of bacteria during the one-week monitoring period. At the one-week point, less than a median of one cfu could be recovered from the lung homogenates.

**Fig 6:**
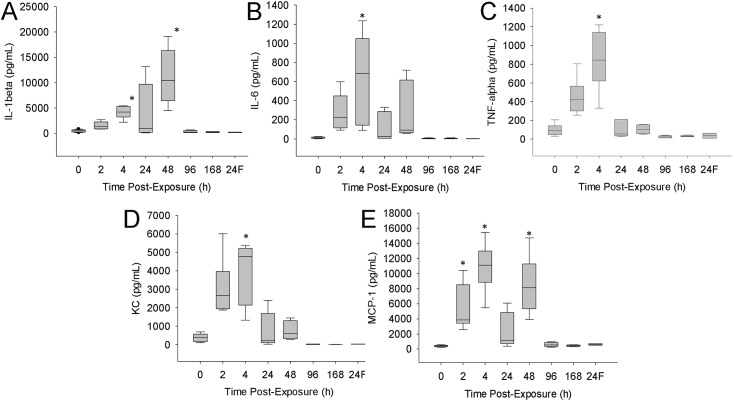
Changes in pulmonary cytokines following exposure. Mice were exposed to 1.25x10^7^ cfu in 25 μL of MBP. At various times post-exposure, a subset of animals was euthanized. Portions of lungs were excised, homogenized, and levels of IL-1β (A), IL-6 (B), TNF-α (C), KC (D), and MCP-1 (E) were measured. The designation ‘24F’ in the x-axis represents 24h post MBP filtrate exposure. The box-whisker plots show the following: The horizontal line within each box represents the median from six animal treatments. Lower and upper boundaries of the boxes represent the 25th and 75th percentiles, respectively. The whiskers represent the 10th and 90th percentiles. Asterisks indicate statistically different values compared to saline-treated controls (p<0.05).

The lungs of these animals were analysed for levels of pro-inflammatory cytokines and chemokines (Fig 6). Of the six cytokines examined, only IL-12(p70) was not present in sufficiently high levels for detection in lung tissue (data not shown). The levels of the IL-6, TNF-α, KC, and MCP-1 were all elevated maximally at 4h post-exposure. IL-1β was maximal at 48h post-exposure. By 96h post-exposure, all cytokines/chemokines were at the level of saline-treated animals. Cytokine/chemokine levels following exposure to MBP filtrate were only evaluated at 24-h post-exposure; indicator levels were not significantly different from that of controls.

**Fig 7:**
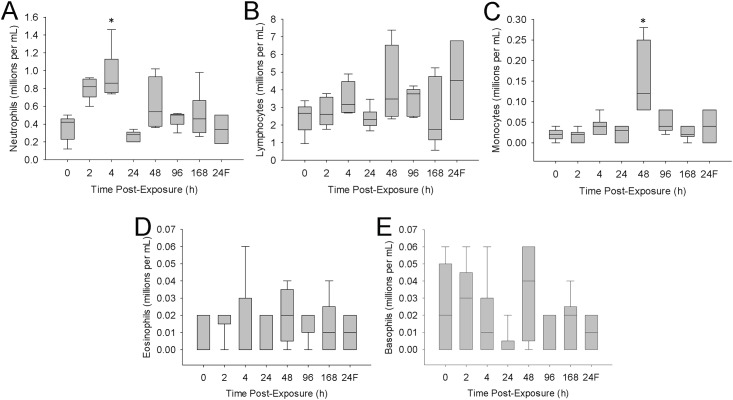
Changes in blood leukocytes following exposure. Mice were exposed to 1.25x10^7^ cfu in 25 μL of MBP. At various times post-exposure, a subset of animals was euthanized. Blood was collected and levels of neutrophils (A), lymphocytes (B), monocytes (C), eosinophils (D), and basophils (E) were measured using a haematology analyser. The designation ‘24F’ in the x-axis represents 24h post MBP filtrate exposure. The box-whisker plots show the following: The horizontal line within each box represents the median from six animal treatments. Lower and upper boundaries of the boxes represent the 25th and 75th percentiles, respectively. The whiskers represent the 10th and 90th percentiles. Asterisks indicate statistically different values compared to saline-treated controls (p<0.05).

As a measure of the systemic effects, whole blood was examined for alterations in leukocyte populations. At four hours post-exposure to the MBP, mice demonstrated significant elevations in blood neutrophils (2-fold of control) (Fig 7A). This was followed by a strong elevation in monocytes (6-fold of control) at 48h post-exposure (Fig 7C). The levels of lymphocytes, basophils and eosinophils were not significantly different from controls during the one week monitoring period (Fig 7B, 7D and 7E). MBP filtrate exposure failed to alter blood leukocyte levels at 24h.

**Fig 8:**
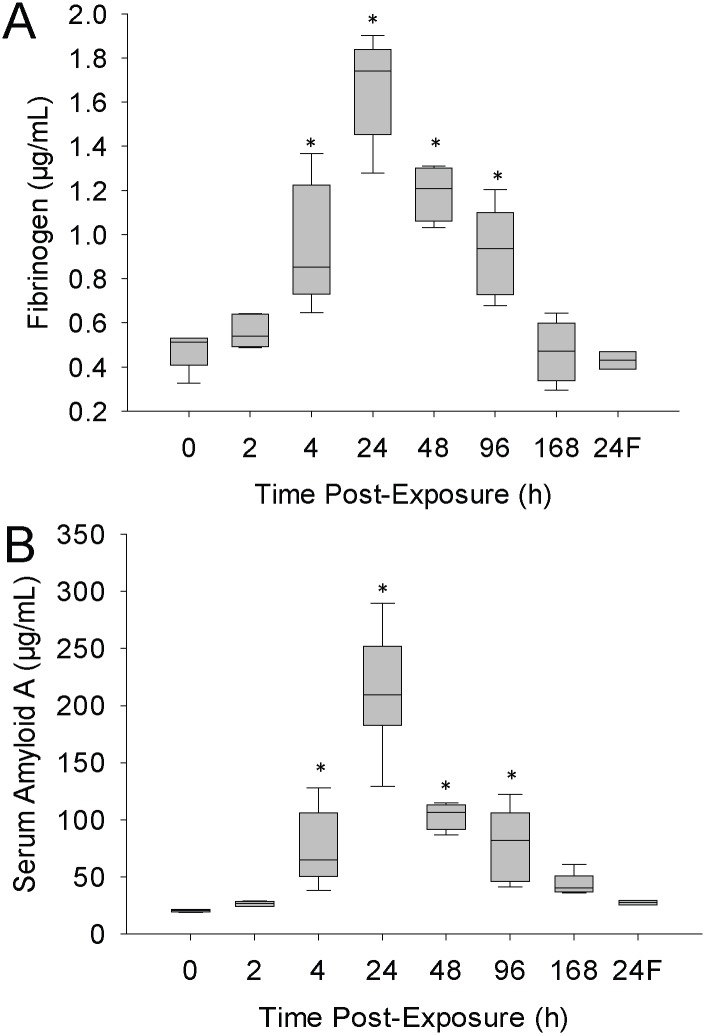
Changes in acute phase response proteins following exposure. Mice were exposed to 1.25x10^7^ cfu in 25 μL of MBP. At various times post-exposure, a subset of animals was euthanized. Blood was collected and levels of fibrinogen (A) and serum amyloid A (B) were measured by ELISA. The designation ‘24F’ in the x-axis represents 24h post MBP filtrate exposure. The box-whisker plots show the following: The horizontal line within each box represents the median from six animal treatments. Lower and upper boundaries of the boxes represent the 25th and 75th percentiles, respectively. The whiskers represent the 10th and 90th percentiles. Asterisks indicate statistically different values compared to saline-treated controls (p<0.05).

Induction of the acute phase response was tested by monitoring levels of fibrinogen and SAA. As seen in Fig 8A and 8B, both indicators were maximally elevated at 24h post-exposure by 3-fold and 10-fold, respectively. Levels of both markers resumed to that of saline at 168h post-exposure. MBP filtrate failed to alter the levels of either marker.

## Discussion

Much effort is being directed towards developing MBPs from complex microbial mixtures and naturally occurring consortia for degradation of environmental contaminants and biosynthesis of compounds. In fact, synthetic biology and sophisticated molecular genetic manipulation methodology has now made it possible that several synthetic microbes could be mixed to generate communities that are able to perform complex processes through multiple coordinated metabolic pathways [18]. However, there is a lack of understanding of the possible toxicological implications associated with human exposure during the manufacture or use of MBPs. Towards developing the necessary baseline knowledge for regulatory guidance, a MBP was selected based on its advertised degradation capacity, reported history of use, and commercial availability.

This MBP product was especially complex and possibly contained a number of pathogenic species, but many other MBPs contain much fewer constituents. Although we described a comprehensive analysis involving multiple methods for microbial identification and both cytotoxicity and murine exposure assessment, implementation of all tests provided here may be unnecessary for development of a systematic testing regime depending on the complexity and initial content analysis of the MBP product. Our results also highlight key considerations for microbial identification (i.e., dynamic state of the MBP, importance of growth dependant and metagenomics methods) and toxicity (i.e., useful *in vitro* markers, limitations of *in vitro* systems for systemic immunological effects, and indicators for assessing effects in mouse models).

The dynamic nature of this living MBP necessitated that products were tested within a narrow duration of time, since our analyses indicated a rapid decline in viability after a few weeks of storage. The results shown here were collected within a four-week span, and demonstrated that a combination of selective media/microbiology, metagenomic and biophysical methods could be used to confidently identify a large number of microbes within the MBP. Using selective and non-selective growth media, the MBP was observed to contain at least 31 culturable species of aerobic bacteria that could be identified using two separate 16S rDNA databases and a FAME library. These species included pathogenic groups of microbes including the *Bacillus cereus* group, *Klebsiella*, and *Enterococcus* (Table 2). Selective media plates allowed for the enrichment of these strains from the MBP, and it was concluded that the overall content of these pathogenic strains is likely low.

**Table 2:** Infections associated with cultivable microbes identified in MBP.

MBP Microbe	Clinical Manifestation	Species Implicated	References
*Arcobacter*	Foodborne gastrointestinal illnesses; diarrhoea	*A*. *butzleri*, *A*. *cryaerophilus*, *A*. *skirrowii*	[24]
*Bacillus*	Gastrointestinal illness; diarrhoea; ocular infections; nosocomial infections; other local and systemic infections	*B*. *cereus*	[25]
*Bacteriodes*	Foodborne gastrointestinal illnesses; diarrhoea; intestinal inflammation	Enterotoxigenic *B*. *fragilis*,	[26]
*Comamonas*	Associated with poly-microbial intra-abdominal infections, peritonitis following appendix rupture; isolated from subcutaneous wound; cellulitis; septicaemia	*C*. *kerstersii*, *C*. *testosteroni*	[27–30]
*Enterococcus*	Urinary tract infections, hepatobiliary sepsis, bacteremia, endocarditis	*E*. *faecalis*, *E*. *faecium*	[31]
*Klebsiella*	Pneumonia; urinary tract infections, nosocomial infections	*K*. *pneumomiae*	[32]
*Proteus*	Rheumatoid arthritis; endopthalmitis, endocarditis	*P*. *mirabilis*	[33–35]

Given the possibility that not all MBP constituents would be culturable, we employed metagenomics approaches for analysis of the product diversity. Both data sets that included randomly sequenced clones of a 16S rDNA library and high-throughput derived16S rDNA sequences derived from pooled PCR amplicons revealed high diversity and species identities distinct from the cultivable species (Fig 1A and 1B). The 16S rDNA library consisted of full length clones of 16S rDNA amplified from the MBP using one primer annealing condition. The high throughput analysis was carried out using a pool of PCR products that were amplified using a temperature gradient, which should have allowed for the improved efficiency of amplification of different targets of rDNA sequences in a sample of metagenomic DNA.

High-throughput sequencing approaches were previously used for analysis of the same product, albeit with short read sequencing of a 16S rDNA variable region and an earlier product lot [12]. Comparing these analyses, the proportion of identified microorganisms and dominant genera were distinctly different. Both studies showed that *Arcobacter* was a major constituent, however, this study revealed other genera of high abundance such as *Proteus*, *Enterococcus* and *Dysgomonas*. As well, potentially pathogenic fungal genera not seen in the earlier product lot were detected including: *Pseudomonadae* and *Aspergillus*. Furthermore, the long read Pacbio CCS data allowed species level resolution for bacteria of potential harm: *Arcobacter cryaerophilus* is associated with human enteritis (as reviewed by Ferreira and colleagues [19]; *Bacteroides coprosuis* and *Clostridium intestinale* are associated with intestinal environments.

The identification of pathogenic bacteria by selective plating and colony identification were important for confirming their viability within the MBP (summarised in Table 2). This was important since the metagenomic DNA sequencing methodology used detected both cultivable and non-cultivable cells. Although not used here, Candelosi and Meschke have reviewed molecular non-culture methods being developed for estimating the viable fraction of microbes derived from metagenomics [20].

Viability aside, the collective data shown here along with that of Samarajeewa and colleagues [12] suggests that lot-to-lot variation is significant. This lack of product consistency is an impediment to product analysis, and has implications for assessing risk. As such, the details for microbial identification (i.e., time of sampling, method of identification, expected species) should be considered to be a standard component for product quality assurance. Furthermore, these studies demonstrate the importance of knowing the shelf-life and storage conditions prior to product use.

We asked the question whether existing antibiotics could be effective in controlling an infection from the different species present within the MBP. For this, two different methods were examined. Commercial agar plates containing selected antibiotics provided some information. However, the limited availability of commercial tests for a large number of antibiotics limited broad screening. Therefore, a broth dilution method was employed to identify the most useful antibiotics (ciprofloxacin, doxycycline, gentamicin, meropenem), and those that were not particularly effective (amoxicillin, aztreonam, ceftazidime, erythromycin, trimethoprim, vancomycin). This method has been used for several individual bacterial species [9,11,15]; however, these data demonstrated that it could also be used to assess the effectiveness of antibiotics against MBPs. For these studies, the MIC of antibiotic was interpreted as the concentration at which there was no microbial MTT formazan. Comparing the broth dilution results to the agar based data demonstrated similar results, except that the quadrant plates showed the appearance of some microorganism in the presence of gentamicin; this was not observed in the broth test. Differences in results between plating and broth dilution methods have been observed previously [21]. Several fungi were observed during selective media growth, so their overall contribution to MTT formazan was also determined by including a replicate plate with amphotericin B in all wells. This showed that under the conditions tested, fungi present in the MBP did not interfere with MIC measurements.

The whole MBP was tested for its toxicity *in vitro* using two commonly used cell lines, HT29 and A549. Experiments done in the absence of antibiotic, 24h growth of microorganisms at all exposure concentrations contributed to MTT formazan production and confounded human cell viability measurements (Fig 3A). An attempt to eliminate this interference was done by including antibiotic during exposure. Low exposure concentrations of 1/320 to 1/80 caused up to a 50% drop in HT29 bioreduction activity, but exposures with ≥1/40 dilution again resulted in bacterial interference. Antibiotic resistance at high bacterial concentrations could have been a result of bacterial aggregation, which prevented contact with antibiotic. Another attempt to eliminate bacterial interference was done by using the trypan blue assay as an alternative to the MTT assay. These results clearly demonstrated a dose-responsive effect of the whole MBP product (Fig 4B). An interesting observation was that there was no A549 toxicity from the acellular filtrate of the MBP at 1/20, but HT29 cells were sensitive to filtrate effects. This material would be expected to contain microbial metabolites, such as hydrogen sulphide, secreted toxins and bacterial cell debris, including cell wall constituents such as LPS shed from bacteria, all of which may contribute to toxicity.

The capacity of exposed cells to signal immune cells was determined by measuring pro-inflammatory cytokines and chemokines released from the cells during exposure. A multiplex system was used to measure multiple markers from a single exposure well. The results showed that for A549, several chemotactic and pro-inflammatory cytokines were significantly elevated during exposure to MBP, but not from filtrate. In contrast, HT29 cells demonstrated only the induction of IL-8, but not other cytokines, with either whole MBP and filtrate (Fig 3D). A possible constituent of the consortium filtrate, LPS, was used as a positive control for chemokine induction in HT29 experiments. It is possible that MBP LPS contributes to induction of IL-8 by HT29.

The suggestion that cellular toxicity and inflammation may result from MBP exposure, led us to investigate the outcome of exposures in a mouse model. The same system used previously with *Bacillus* and *Pseudomonas* species was employed here [8,11]. When administered by endotracheal instillation, it was demonstrated that the aerobic culturable MBP contents were rapidly cleared from the lung within the first 48h (Fig 5). At one-week post-exposure, residual microorganisms were not observed. This rapid clearance of microbes has been observed in previous studies [8,11]. However, whether other particulate matter (e.g., dead bacteria, cellular debris or facultative anaerobes) remained in the tissue and contributed to virulence is not known.

The lungs were further analysed for evidence of inflammation. The levels of several pro-inflammatory cytokines and chemotactic markers were observed to increase between 4 and 48h post-exposure. In particular, IL-1β, IL-6 and TNF-α were all significantly, but transiently elevated. These cytokines are strongly associated with inflammation and induction of systemic immune responses. As well, KC and MCP-1 are both chemotactic for neutrophils and monocytes, respectively, and contribute to the recruitment of leukocytes to the tissue.

Blood leukocyte levels were measured to determine if there was an accompanying immune effect at the periphery. Fig 7 shows that there was an early (4h) elevation in the levels of neutrophils and a late (48h) elevation in monocytes. Neutrophils typically appear at infection sites to both sequester and kill invading microorganisms, simultaneously causing collateral damage to surrounding tissue [22]. In mice, blood monocytes differentiate into tissue macrophages and may act to repair damaged tissue and remove debris. The function of tissue macrophage in tissue repair and regeneration has been reviewed previously [23].

Since IL-1β, IL-6 and TNF-α were all elevated, the levels of blood acute phase response proteins, fibrinogen and SAA, were measured. These proteins produced by the liver are evidence of a systemic response to infection or trauma, which functions to promote healing and restore homeostasis. Data presented in Fig 8 show that both markers were significantly elevated at 4 to 96h post-exposure, with maximal blood levels at 24h.

The MBP filtrate was found to be toxic towards HT29 cells, and was capable of inducing significant levels of IL-8. However, in our mouse exposure model, there was no observed effect with any of the indicators examined. A549 exposure more closely resembled results from the murine experiment, which may demonstrate that lung epithelial cells are irresponsive compared to colonic epithelial cells. It should also be noted that during the murine studies, effects from MBP filtrate were only evaluated at 24-post exposure, and this time-point might not have been optimal for observing toxicological effects. Although the composition of the MBP filtrate is unknown, it may contain carry-over from industrial scale-up culture, as well as microbial metabolites and dissociated structural components of microbial cells, such as LPS. It is possible that IL-8-inducing activity of the MBP filtrate was driven by these components, but were easily sequestered, cleared or inactivated from the mouse lung quickly after exposure. This elimination system would clearly not be present *in vitro*. Alternatively, there may be a tissue specificity associated with the toxicity of the filtrate, which was not tested here.

**Fig 9:**
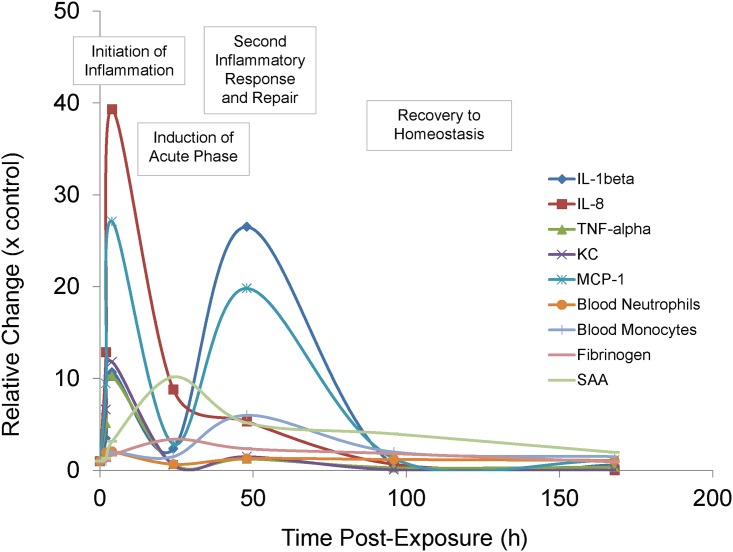
Summary of immunological markers during exposure. Markers measured during experiments were expressed relative to control values. Data points represent median values obtained from six replicate mice. Error bars have been omitted for clarity, but the variability of this data is represented in Figs 4, 5 and 6.

A summary of the events observed is presented in Fig 9. Four steps can be envisaged from the data presented. Initiation of inflammation (2 to 4h) was characterised by elevation in the pro-inflammatory cytokines IL-1β, IL-6, and TNF-α, which in turn activated the production of APR proteins, fibrinogen and SAA (24h). Early elevation of the KC at 2 to 4h caused an elevation of blood neutrophils at 4h. A second inflammatory response (48h) was observed, which was characterised by a strong second IL-1β and MCP-1 response. At this time, blood monocyte levels were also notably higher, which may be indicative of the initiation of tissue repair. The final phase was that of all markers returning to homeostatic levels.

The data presented here demonstrate that MBPs can be complex in their composition, including both intentional and contaminating constituents. Commercial products should be scrutinized to guarantee their contents. Further, the model MBP used here had the capacity to induce transient, but significant local inflammation and systemic responses during exposure. Multiple levels of evidence dictate that caution should be used during industrial scale production and environmental dissemination of the product to avoid human exposure.
